# *Escherichia coli* Uses Separate Enzymes to Produce H_2_S and Reactive Sulfane Sulfur From L-cysteine

**DOI:** 10.3389/fmicb.2019.00298

**Published:** 2019-02-20

**Authors:** Kai Li, Yufeng Xin, Guanhua Xuan, Rui Zhao, Huaiwei Liu, Yongzhen Xia, Luying Xun

**Affiliations:** ^1^State Key Laboratory of Microbial Technology, Shandong University, Qingdao, China; ^2^College of Life Sciences, Qufu Normal University, Qufu, China; ^3^School of Molecular Biosciences, Washington State University, Pullman, WA, United States

**Keywords:** L-cysteine, hydrogen sulfide, reactive sulfane sulfur, antioxidant, 3-mercaptopyruvate sulfurtransferase

## Abstract

Hydrogen sulfide (H_2_S) has been proposed to have various physiological functions, and it may function through reactive sulfane sulfur. Since the two sulfur forms often coexist, they are normally considered interchangeable. Here, we characterized the production of H_2_S and reactive sulfane sulfur in *Escherichia coli* MG1655 and found that they are not readily interchangeable. They are primarily produced from L-cysteine via different enzymes. L-Cysteine desulfhydrases consumed L-cysteine and directly generated H_2_S. The produced H_2_S was mainly lost through evaporation into the gas phase, as *E. coli* does not have enzymes that easily oxidize H_2_S to reactive sulfane sulfur. L-Cysteine desulfhydrases were also responsible for the degradation of exogenous L-cysteine, which is toxic at high levels. Conversely, L-cysteine aminotransferase and 3-mercaptopyruvate sulfurtransferase sequentially metabolized endogenous L-cysteine to produce cellular reactive sulfane sulfur; however, it was not a major route of H_2_S production during normal growth or during the metabolism of exogenous L-cysteine by the resting cells. Noticeably, the 3-mercaptopyruvate sulfurtransferase mutant contained less reactive sulfane sulfur and displayed a greater sensitivity to H_2_O_2_ than did the wild type. Thence, reactive sulfane sulfur is likely a common cellular component, involved in protein sulfhydration and protecting cells from oxidative stress.

## Introduction

H_2_S may protect bacterial cells from oxidative stress (Shatalin et al., 2011) and have physiological roles in cellular signaling through protein sulfhydration (Paul and Snyder, 2012; Giedroc, 2017), in which a sulfur atom is added to a cysteinyl thiol in a target protein to form a hydropersulfide (protein-SSH). Sulfhydration is involved in cellular signaling and in modulating enzyme activity (Toohey, 1986; Wang, 2010; Kimura, 2014). However, the direct involvement of H_2_S in protein sulfhydration has been questioned, as it does not have the ability to directly react with a thiol group (-SH) (Toohey, 2011; Pan and Carroll, 2013; Yadav et al., 2016). On the other hand, reactive sulfane sulfur (RSSH, RSS_n_H, n ≥ 1; HSS_n_H and RS(S)_n_SR; n ≥ 1), which does not include the insoluble elemental sulfur, can react with protein thiols to generate protein-SSH (Toohey, 2011; Ida et al., 2014). There are appreciable amounts (>100 μM) of reactive sulfane sulfur in the plasma, cells, and tissues of mammals (Ida et al., 2014). Thus, it is unresolved whether H_2_S or reactive sulfane sulfur is mainly responsible for protein sulfhydration and resistance to oxidative stress in live cells (Toohey, 2011; Ida et al., 2014).

H_2_S and reactive sulfane sulfur often coexist, making it difficult to precisely distinguish their production and function. H_2_S can lead to the production of reactive sulfane sulfur via chemically reacting with a disulfide bond and a sulfenic acid (Nagy, 2015) or via oxidation by sulfide:quinone oxidoreductases (SQR) and flavocytochrome c-sulfide dehydrogenases (FCSDs) (Libiad et al., 2014; Shen et al., 2016; Lü et al., 2017). Reactive sulfane sulfur can also be generated from sulfur-containing amino acids by several enzymes. Cystathionine β-synthase (CBS) and cystathionine γ-lyase (CSE) can produce CysSSH from cystine (Cys-SS-Cys) (Ida et al., 2014), but the contribution of these reactions to cellular reactive sulfane sulfur may be limited, as the concentration of reduced thiols is significantly higher than that of disulfides in the reducing cytoplasmic milieu (Yadav et al., 2016). Cysteinyl-tRNA synthetase may directly use L-cysteine to produce Cys-SSH (Akaike et al., 2017). Further, L-cysteine aminotransferase (CAT) can convert L-cysteine to 3-mercaptopyruvate (Nagahara and Sawada, 2006); mercaptopyruvate sulfurtransferase (MST) abstracts the sulfur of 3-mercaptopyruvate to generate a persulfide at its active site (MST-SSH) (Toohey, 2011; Yadav et al., 2013). On the flipside, the various forms of reactive sulfane sulfur can be reduced by cellular thiols such as GSH, thioredoxin, and glutaredoxin to product H_2_S (Mikami et al., 2011; Yadav et al., 2013; Dóka et al., 2016). However, a systematic investigation on the interchangeability of H_2_S and sulfane sulfur in live cells has not been investigated.

*Escherichia coli* MG1655 contains CAT/MST (Nagahara and Sawada, 2006; Shatalin et al., 2011) and at least six enzymes (CysK, CysM, MetC, TnaA, MalY, and YhaM) with L-cysteine desulfhydrase (CD) activity (Awano et al., 2005; Shimada et al., 2016). CDs decompose L-cysteine into pyruvate, ammonium, and H_2_S. Since *E. coli* does not have CBS, CSE, SQR, and FCSDs, it is a good system to evaluate the roles of CDs and MST in the production of H_2_S and reactive sulfane sulfur in live cells. Here, we characterized their roles, evaluated the interchangeability of H_2_S and reactive sulfane sulfur, and investigated whether H_2_S or reactive sulfane sulfur is the major antioxidant in *E. coli*.

## Materials and Methods

### Bacterial Strains, Plasmids, Gene Deletion, Cloning, and Growth Conditions

All deletions in *E. coli* MG1655 were performed by using a reported one-step deletion method (Datsenko and Wanner, 2000). For complementation or gene expression, the target genes were amplified by PCR, and the PCR products were cloned into linearized pBBR1MCS2 or pET30-LIC by using the In-Fusion HD cloning kit (Clontech, United States). All bacterial strains, plasmids and recombinant cells are listed in Supplementary Table S1, and all primers are given in Supplementary Table S2. *E. coli* was cultured in LB medium at 37 or 25°C with shaking (200 rpm). Ampicillin (100 mg/L) or kanamycin (50 mg/L) was added as needed.

### Sulfur-Containing Compounds Metabolize by *E. coli*

Overnight culture of *E. coli* MG1655 was inoculated in fresh LB medium to OD_600__nm_ of 0.05. The cells were cultured to about OD_600__nm_ of 4.0, harvested, washed and resuspended in 50 mM Tris-HCl buffer, pH 7.6, at OD_600__nm_ of 2.0. Appropriate sulfur-containing compounds were added to a final concentration of 100 μM to the resting cells to initiate the reaction. Glucose was also added to 1% to provide a reducing power. The production of H_2_S and reactive sulfane sulfur was detected.

### Recombinant Protein Purification

*Escherichia coli* BL21(DE3) cells with the expression plasmid pET-TnaA, pET-MalY, pET-CysK, pET-CysM, pET-YhaM, pET-CAT, or pET-MST were inoculated in LB medium and cultured with shaking to OD_600__nm_ of 0.6 at 25°C, and then isopropyl β-D-thiogalactoside (IPTG) was added to a final concentration of 0.5 mM. The cultures were further cultured for about 10 h. Cells were harvested by centrifugation and disrupted by a pressure cell homogenizer (Stansted Fluid Power LTD, United Kingdom, SPCH-18) at 4°C in buffer I (20 mM Tris-HCl, 0.5 M NaCl, 20 mM imidazole, pH 8.0). The lysate was centrifuged at 12,500 × *g* for 10 min to remove cell debris 4°C. The target protein was purified by using nickel-nitrilotriacetate agarose (Qiagen, Shanghai, China), according to the supplier’s recommendations. The buffer was exchanged to 20 mM sodium phosphate buffer (pH 7.6) and then 50% glycerol was added to give a final concentration of 10% before storage at -80°C.

### Enzyme Activity Assays

The activity of CDs and CAT/MST for the degradation of L-cysteine was assayed. The assay was routinely performed at 25°C. The reaction was carried out in 3 mL of 50 mM Tris-HCl buffer, pH 7.6, in the test tube (1.5 × 10 cm), containing 50 μM pyridoxal phosphate, 1.0 mM L-cysteine, and 1.0 mM α-KG (only for CAT/MST reactive mixture). The purified CD or CAT/MST (both purified CAT and MST) was added at the final concentration of 1 μM to initiate the reaction. After 1 h incubation, the production of H_2_S were detected by using a reported monobromobimane (mBBr) method (Kolluru et al., 2013) and reactive sulfane sulfur produced in the reaction was detected with SSP4 (Yadav et al., 2016; Bibli et al., 2018).

### Detection of H_2_S, Sulfide, Sulfite, and Thiosulfate

The H_2_S generation by *E. coli* was monitored with both lead acetate [Pb(Ac)_2_] paper strips (Shatalin et al., 2011) and the mBBr method. Briefly, a [Pb(Ac)_2_] paper strip with lead acetate was affixed to the inner wall of a glass tube (1.5 × 20 cm) containing 5.0 mL of the culture. H_2_S was evaporated into the gas phase and reacted with Pb(II) to form dark PbS stains, which were measured via densitometer and evaluated by using a standard curve. To obtain the standard curve, various concentrations NaHS were added to 5 mL of LB medium and after incubation at 37°C with shaking for 12 h, the darkness on the paper strips were measured via densitometer (Chen et al., 2018). Sulfide (H_2_S, HS^-^, and S^2-^), sulfite, and thiosulfate in the liquid phase were detected by using the mBBr method (Kolluru et al., 2013). Briefly, 50 μL of a sample was reacted with 5 μL of 25 mM mBBr at room temperature for 30 min in the dark, and an equal volume of 10% acetic acid in acetonitrile was added. The precipitated proteins were removed by centrifugation at 12,500 × *g* for 2 min. Then, 20 μL of the supernatant was analyzed by using HPLC (LC-10AT, Shimadzu) with a fluorescence detector (Xin et al., 2017).

### Detection of Reactive Sulfane Sulfur

SSP4 was used to detect reactive sulfane sulfur in the enzyme reaction mixtures. 10 mM SSP4 stock solution in acetonitrile was added to a reaction mixture to a final concentration of 10 μM, and the sample was mixed and incubated in the dark at 37°C for 10 min. The fluorescence was detected by using the Synergy H1 microplate reader with excitation of 482 nm and the emission of 515 nm.

Reactive sulfane sulfur produced by *E. coli* cells in the Tris-HCl buffer during the metabolism of 100 μM sulfur-containing substances was detected by adding 10 μM SSP4 and 0.5 mM CTAB in the reaction mixture. The sample was incubated in the dark at 37°C with shaking for 20 min, and the cells were harvested by centrifugation and washed twice with phosphate buffer saline (PBS). The fluorescence of the resuspended cells (OD_600__nm_ of 0.2) in PBS was detected by using the Synergy H1 microplate reader.

Reactive sulfane sulfur in the supernatant of lysed *E. coli* cells was also detected. The cells were harvested, washed and resuspended in 50 mM anoxic Tris-HCl buffer, pH 7.6, and disrupted by a pressure cell homogeniser. The lysate was centrifuged at 12,500 × *g* for 10 min to remove cell debris at 4°C. The sulfane sulfur in the supernatant was measured with SSP4 as above. The fluorescence values were expressed as the intensity per mg of protein.

### Analysis of MP Metabolites

*Escherichia coli* MG1655, its mutant *E. coli* Δ*sseA*, and its recombinant cells were cultivated in LB medium at 37°C. When OD_600__nm_ reached to 0.6∼0.8, 0.5 mM IPTG was added, and the cells were further cultivated for 12 h at 37°C. The cells were harvested, washed and resuspended at OD_600__nm_ of 2 in 50 mM Tris-HCl buffer, pH 7.6. Ten mL of the cells suspension was transferred to a 50-mL centrifuge tube, and 1% glucose and 0.2 mM MP were added to initiate the reaction. The tube was loosely capped and incubated at 37°C with shaking. The produced sulfide, sulfite, and thiosulfate were analyzed by the mBBr method at various time intervals (Kolluru et al., 2013).

### Measuring L-cysteine Degradation by *E. coli* Whole Cells

*Escherichia coli* MG1655 and its mutants were cultured, harvested, washed and resuspended in 50 mM Tris-HCl buffer, pH 7.6, at OD_600__nm_ of 2. Glucose was added to 1%, and L-cysteine and α-KG were added to1 mM to initiate the reaction. Ellman’s reagent (DTNB) was used to detect L-cysteine in the supernatant (Riener et al., 2002). Briefly, 1 mL of a sample was added to 99 μl of the DTNB reagent, mixed, and incubated at 37°C for 10 min. The absorbance at 412 nm was measured to determine L-cysteine contents with a standard curve.

### Generation of Survival Curves After H_2_O_2_ Treatment

Overnight cultures of *E. coli* strains were transferred in LB medium at 1% inoculum and grown to OD_600__nm_ of 1 (37°C, 200 rpm). Cells were then treated with 2.5 mM H_2_O_2_ at room temperature for 20 min without shaking and diluted to stop the treatment. The diluted cells were spread on LB medium agar plates and incubated at 37°C for 16∼18 h. Cell survival was determined by counting colony forming units (CFU). The averages of three samples and standard deviation were reported.

### Analysis of MST in Sequenced Bacterial Genomes

Bioinformatics analysis was done as reported (Lü et al., 2017; Xia et al., 2017) with minor modification. Briefly, a microbial genomic protein sequence set from NCBI updated until November 11, 2017 was downloaded for MST search. The query sequences of MST were confirmed MSTs (Nagahara and Sawada, 2006; Westrop et al., 2009; Wu et al., 2015) and were used to search the database by using Standalone BLASTP algorithm with conventional criteria (*e*-value ≤ 1e-10, coveryage ≥ 45%, identity ≥ 35%) to obtain MST candidates from a total of 8671 bacterial genomes. Then the candidates performed multiple sequence alignments to exclude sequences by Clustal Omega^1^ that are not MST based on conserved cysteine residues at the catalytic site (Yadav et al., 2013). The candidates combined with the seed MSTs for phylogenetic tree analysis by using ClustalW for alignment and MEGA version 7.0 to generate the tree by using neighbor-joining with a pairwise deletion, p-distance distribution, and bootstrap analysis of 1,000 repeats as the parameters (Kumar et al., 2016). The candidates that were in the same clade with the seed MSTs were picked up for further analysis. The identified MST sequences were separately grouped into unique groups by using the CD-HIT program with a threshold of 90% sequence identity or better. Since rhodaneses display clear sequence homology with MST (Nagahara et al., 1995), five published rhodanese sequences were also collected (Cheng et al., 2008) and grouped into unique groups as outgroups. One representative sequence from each unique group was selected and used to build a phylogenetic tree (MEGA Version 7.0).

## Results

### CDs and CAT/MST in L-cysteine Degradation and H_2_S Accumulation

**Figure 1:**
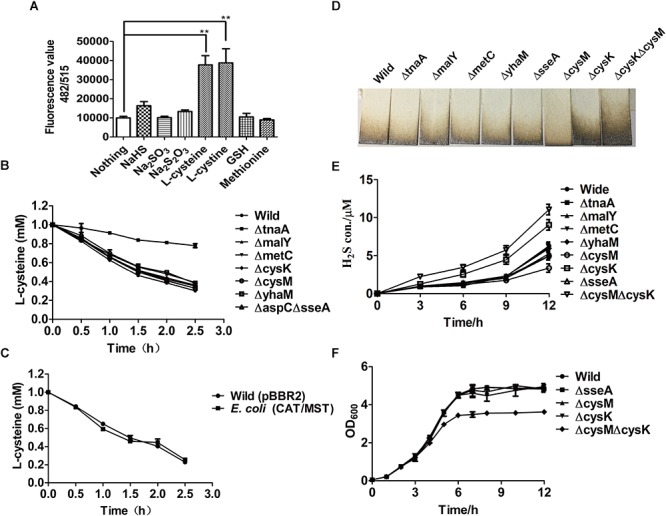
The production of H_2_S and reactive sulfane sulfur from L-cysteine by the resting cells of *E. coli* MG1655 and its mutants. **(A)** Reactive sulfane sulfur as detected with SSP4 within the wild type after 30-min incubation with various substrates. **(B)** Effects of the deletion of CDs or CAT/MST on L-cysteine degradation. **(C)** The effect of CAT and MST overexpression on L-cysteine degradation. **(D)** Pb(Ac)_2_ paper strips detected the release of H_2_S into the gas phase by *E. coli* MG1655 and its mutants grown in LB medium for 12 h. **(E)** Sulfide was quantified in the supernatant of *E. coli* MG1655 and mutants grown in LB medium. **(F)** The growth curve of *E. coli* MG1655, *E. coli*Δ*sseA* (ΔsseA), *E. coli*Δ*cysM* (ΔcysM), *E. coli*Δ*cysK* (ΔcysK), and *E. coli*Δ*cysK*Δ*cysM* (ΔcysKΔcysM) in LB medium. Overnight cultures of *E. coli* MG1655 and mutant strains were inoculated in fresh LB medium to OD_600__nm_ of 0.05, and the growth was monitored via OD_600nm_ at 1-h intervals.

*Escherichia coli* MG1655 was grown in Lysogeny broth (LB) medium, harvested, and resuspended in 50 mM Tris buffer (pH 7.6) at OD_600__nm_ of 2.0. The resting cells were then tested to produce H_2_S and reactive sulfane sulfur from various sulfur-containing compounds. L-Cysteine and L-cystine were the most suitable substrates for the production of H_2_S (Supplementary Figure S1) and reactive sulfur sulfane compared with other organic (GSH and methionine) and inorganic (Na_2_SO_3_ and Na_2_S_2_O_3_) substances (Figure 1A). When GSH was tested, the cells did not generate detectable H_2_S in 30 min but produced H_2_S in 1 h of incubation (Supplementary Figures S1, S2A). Methionine did not have a significant effect on the production of H_2_S and reactive sulfane sulfur even after 12 h incubation (Figure 1 and Supplementary Figure S1A). *E. coli* released approximately 100 μM H2S after growing in LB medium for 12 h, and the inclusion of methionine up to 10 mM did not affect the H_2_S production or the cell growth (Supplementary Figure S2B).

To assess the contribution of CAT/MST and CDs in L-cysteine degradation and H_2_S production by *E. coli* MG1655 under aerobic conditions when culture in LB medium, a series of mutants were constructed and tested. The resting cells of *E. coli* strains in the Tris buffer at OD_600nm_ of 2.0 were used to degrade 1.0 mM L-cysteine. After 2.5 h incubation, *E. coli* MG1655 and its mutants Δ*tnaA*, Δ*malY*, Δ*metC*, Δ*cysK*, Δ*cysM*, Δ*yhaM*, and Δ*aspC*Δ*sseA* degraded 69.9, 22.1, 61.2, 65.0, 63.9, 62.1, 66.9, 67.2% of the added L-cysteine, respectively (Figure 1B). The results indicated that TnaA plays a major role in the degradation of added L-cysteine while others including the CAT/MST pathway have limited contribution to the degradation. The overexpression of CAT/MST [*E. coli* (CAT/MST)] did not increase the rate of L-cysteine degradation compared with the wild type (Figure 1C).

The effect of the gene deletions on H_2_S accumulation during growth in LB medium was also tested (Figure 1D). The wild type, Δ*tnaA*, Δ*malY*, Δ*metC*, and Δ*sseA* all released around 100 μM H_2_S after 12 h of incubation; however, Δ*cysK* accumulated approximately double H_2_S and Δ*cysM* produced only roughly two-thirds H_2_S compared with wild type (Figure 1D). The double mutant *E. coli*Δ*cysK*Δ*cysM* accumulated more than twice of H_2_S compared with wild type (Figure 1D), indicating that the lack of H_2_S consumption by CysK and CysM contributes to its accumulation. Sulfide in the culture supernatant was further detected with the mBBr method, and the results also showed that Δ*tnaA*, Δ*malY*, Δ*metC*, and Δ*sseA* produced similar amounts of sulfide as the wild type, but Δ*cysK* and Δ*cysK*Δ*cysM* produced more sulfide (Figure 1E). The deletion of single genes did not affect the growth of the mutants in LB medium (Figure 1F). The double mutant *E. coli*Δ*cysK*Δ*cysM* had the same initial growth rate, but had a reduced final yield (Figure 1F). Collectively, the data suggest that *E. coli* produces H_2_S during growth in LB medium and uses some of the produced H_2_S to produce L-cysteine by CysK and CysM.

### Different Mechanisms of H_2_S Generation by CDs and CAT/MST

**Figure 2:**
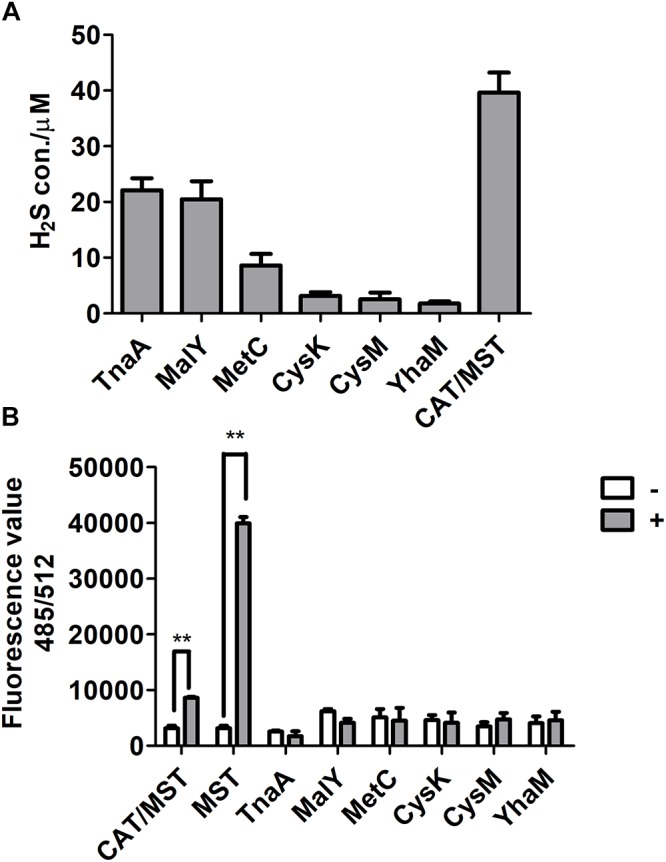
The production of H_2_S and reactive sulfane sulfur from L-cysteine by purified enzymes. **(A)** The purified CD or CAT/MST (CAT and MST) at the final concentration of 1 μM was used to metabolism 1 mM L-cysteine. After 1 h, the production of sulfide was quantified with the mBBr method. **(B)** Simultaneously, the production of reactive sulfane sulfur with (**+**) or without (-) L-cysteine or MP for MST were monitored by using SSP4. Data were presented as the average ± SD from three independent experiments. *T* tests were performed to calculate the *p*-values, and double asterisks indicate statistically significant difference (^∗∗^*p* < 0.01).

The purified CDs and CAT/MST all used L-cysteine and released H_2_S (Supplementary Figure S3 and Figure 2A). However, CysK and CysM had very low activity, suggesting they may not contribute to L-cysteine metabolism *in vivo*. The production of reactive sulfane sulfur during L-cysteine degradation by these enzymes was also tested with SSP4. CDs did not produce reactive sulfane sulfur, while CAT and MST produced significant amounts of reactive sulfane sulfur during L-cysteine metabolism in the presence of the co-substrate α-KG (Figure 2B). Thus, CDs did not generate reactive sulfane sulfur as an intermediate during L-cysteine degradation, while CAT/MST did.

### Reactive Sulfane Sulfur Is the Product of MST *in vitro* and *in vivo*

**Figure 3:**
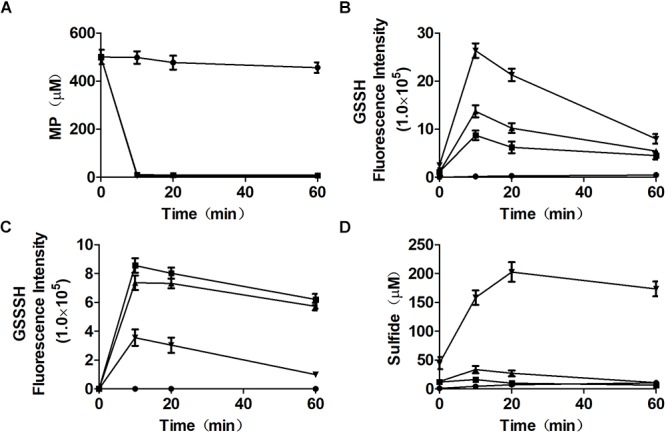
Reactive sulfane sulfur is the direct product of the CAT/MST pathway *in vitro*. MST catalyzes the reaction of MP with different concentrations of GSH *in vitro*. MST catalysis of 500 μM MP with 250 μM (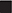), 500 μM (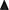), or 2.0 mM (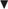) GSH; control without MST (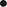), containing MP (500 μM) and GSH (500 μM). The substrate **(A)** MP, or products **(B)** GSSH, **(C)**, GSSSH, and **(D)** Sulfide in solution were analyzed via the mBBr method.

To clarify that MST consumed MP and produced reactive sulfane sulfur, purified MST was used to metabolize MP in the presence of GSH (Figure 3A–D). With the increase of GSH concentrations in the reaction mixture, GSSH also increased (Figure 3B), but GSSSH decreased (Figure 3C). Sulfide was almost not produced when GSH was less than MP in the reaction mixture (Figure 3C). GSSH and GSSSH reached to the maximum at 10.0 min of the reaction (Figure 3B), while the maximal amount of sulfide was achieved at 30 min (Figure 3D). Thus, MST converted MP mostly to reactive sulfane sulfur (Figure 3B,C), and the latter was reduced by GSH to release sulfide in a delayed reaction (Figure 3D). However, the chemical reaction was concentration-dependent and was slower than the enzymatic reaction.

**Figure 4:**
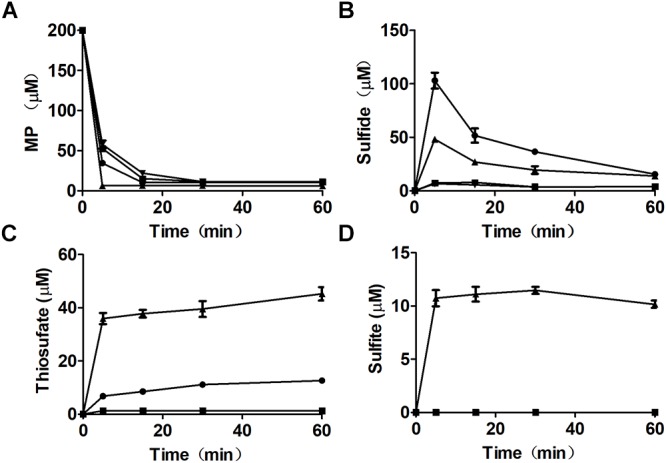
MP metabolism by recombinant *E. coli* cells. Resting cells of *E. coli* (pBBR2) (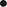), *E. coli* (AtBlh) (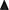), *E. coli* Δ*sseA* (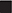), and *E. coli* Δ*sseA* (AtBlh) (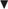) at OD_600nm_ of 2 in 50 mM Tris-HCl buffer, pH 7.6, with 1% glucose was used to degrade 200 μM MP. **(A)** MP, **(B)** sulfide, **(C)** thiosulfate, and **(D)** sulfite were analyzed.

**Figure 5:**
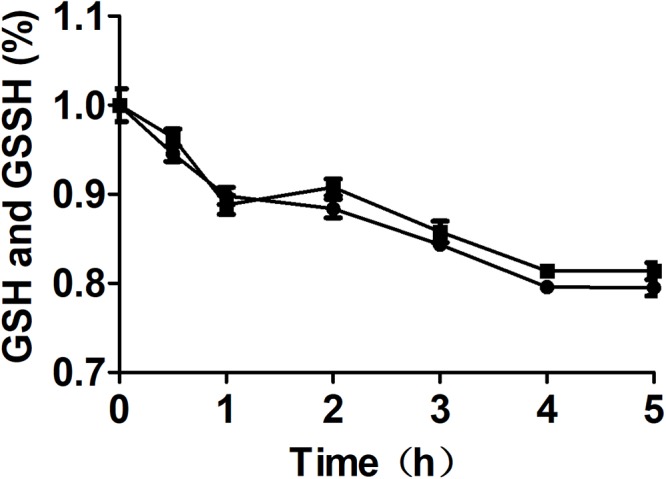
The effect of MST on the stability of GSSH in the presence of GSH. The reaction mixture contained 0.5 mM GSSH, 1.0 mM GSH with (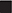) or without (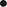) 2 μM MST. The decay of total thiol (GSSH and GSH) was analyzed by using Ellman’s reagent.

Mutant and recombinant *E. coli* strains were also used to metabolize MP. MP is unstable in neutral solutions (Cooper et al., 1982), and it rapidly disappeared even in a reaction with the MST mutant *E. coli* Δ*sseA* and *E. coli* Δ*sseA* (AtBlh) (Figure 4A), but its product was unknown, as sulfide, thiosulfate, and sulfite were not detectable (Figure 4B–D). The wild type with the cloning vector pBBR2 rapidly metabolized 200 μM MP to 105.0 ± 7.5 μM sulfide (Figure 4B) and 7.0 ± 0.86 μM thiosulfate in 5 min, and sulfide in the solution was then rapidly decreased likely due to evaporation with shaking (Figure 4C). To test whether GSSH was generated *in vivo*, persulfide dioxygenase gene (blh) from *Agrobacterium tumefaciens* str. C58 was cloned into *E. coli* MG1655 [*E. coli* (AtBlh)], and recombinant cells rapidly metabolized 200 μM MP to 38.2 ± 2.1 μM sulfide, 55.9 ± 5.7 μM thiosulfate and 10.7 ± 1.5 μM sulfite (Figure 4B–D). These results suggest that GSH can act as a receptor for reactive sulfane sulfur during the MST reaction to produce GSSH under physiological conditions, and GSSH reacts with small thiols such as GSH to release sulfide. MST did not speed up the reaction of GSSH and GSH (Figure 5), suggesting the reaction occurs non-enzymatically.

### Reactive Sulfane Sulfur Protects *E. coli* From H_2_O_2_ Toxicity

**Figure 6:**
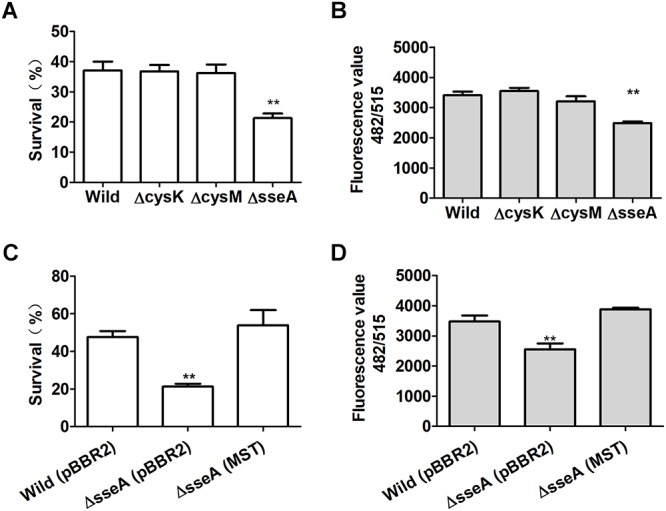
Reactive sulfane sulfur produced by MST protects *E. coli* from H_2_O_2_. **(A)** The survival of H_2_S underproducing (ΔcysM), overproducing (ΔcysK) or normal (ΔsseA and the wild type) strains under H_2_O_2_ challenge. **(B)** The intracellular reactive sulfane sulfur content as measured with SSP4 per mg of protein in the supernatant of the lysed *E. coli* strains. **(C)** The survival of the complemented *E. coli* Δ*sseA* (MST) under H_2_O_2_ challenge. **(D)** The intracellular reactive sulfane sulfur content in the supernatant of the lysed *E. coli* Δ*sseA* (MST). Data were presented as average ± SD of three independent experiments. *T*-tests were performed to calculate the *p*-values, and double asterisks indicate statistically significant difference (^∗∗^*p* < 0.01).

*Escherichia coli* and it mutants were grown in LB medium to OD_600__nm_ of 1. The wild type, ΔcysM, ΔcysK, and ΔsseA produced and released 0.94, 0.9.6, 1.25, and 0.98 μM sulfide into the culture supernatants, respectively (Figure 1E). At OD_600__nm_ of 1, they were also challenged with H_2_O_2_. *E. coli* Δ*sseA* exhibited a much greater sensitivity to H_2_O_2_ than did the wild type, while *E. coli*Δ*cysK* and *E. coli* Δ*cysM* showed similar sensitivity to H_2_O_2_ (Figure 6A). Compared with the wild type, the intracellular content of reactive sulfane sulfur per mg of protein in the supernatant of *E. coli* Δ*sseA* lysate was clearly reduced (Figure 6B). The complemented mutant *E. coli* Δ*sseA* (MST) recovered the content of reactive sulfane sulfur and the survival rate when treated with H_2_O_2_ (Figure 6C,D). Thus, MST contributes to the production of cellular reactive sulfane sulfur that confers resistance to H_2_O_2_.

### Distribution of MST in Bacteria

**Figure 7:**
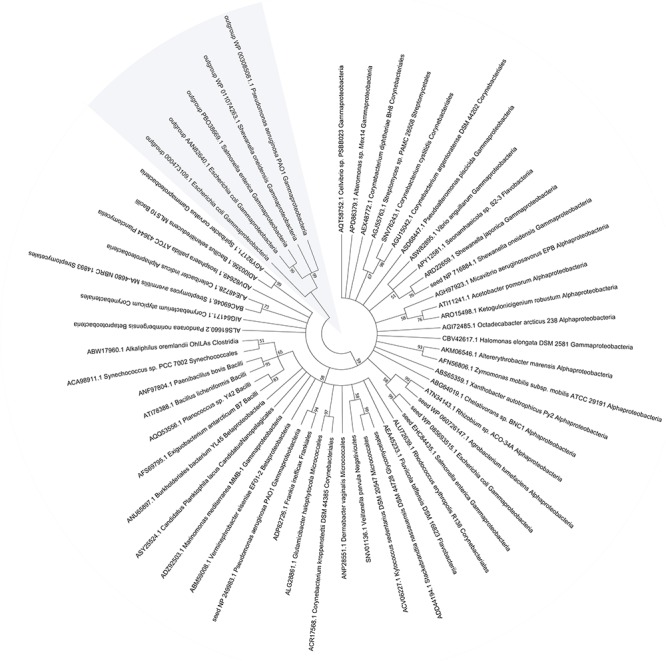
The phylogenetic tree and distribution of MST in bacteria. The representative MST sequences were used for phylogenetic tree construction with reported seed sequences.

To assess the importance of MST, we evaluated the conservation and distribution of MST in bacteria. Five confirmed MSTs (Spallarossa et al., 2004; Nagahara and Sawada, 2006; Mikami et al., 2011) were used as seed sequences to BLAST a microbial genomic protein sequence set of 8671 bacterial genomes (NCBI updated until November 11, 2017). Our result showed that 48.2% the sequenced genomes contained MST, 97% of which had only one MST; most of MSTs (89.2%) were distributed in six classes: Gammaproteobacteria (45.4%), Betaproteobacteria (18.3%), Alphaproteobacteria (12.3%), Bacilli (7.3%), Corynebacteriales (6.0%), and Streptomycetales (1.9%) (Supplementary Table S3). We also searched the conserved domain (CDD) of the MSTs at the NCBI website and found that all MSTs contained the CDD COG2897 belonging to the cl25399 family. While the five rhodaneses that were used as the outgroup references of the phylogenetic tree contained the CDD PRK00162 belonging to the cl00125 family (Figure 7). The phylogenetic tree showed that MSTs were not grouped into distinct branches, corresponding to their wide distribution.

## Discussion

L-Cysteine is the major source of H_2_S and reactive sulfane sulfur for *E. coli*, while methionine is not (Figure 1A and Supplementary Figure S1). Although thiosulfate can be used directly by CysM for the production of L-cysteine (Zhao et al., 2006), the resting *E. coli* cell did not use it to contribute to H_2_S production in the presence of glucose. It did not use the assimilatory sulfite reduction pathway to generate extra H_2_S, either, as sulfite did not stimulate H_2_S production (Supplementary Figure S1). *E. coli* mainly used CDs and CAT/MST to metabolize L-cysteine (Figure 2–4).

CDs convert L-cysteine to H_2_S, while the CAT/MST pathway produces reactive sulfane sulfur, due to different catalytic mechanisms. CDs catalyze L-cysteine metabolism through a mechanism of α,β-elimination, β-replacement, or α-hydrogen exchange with the formation of a double-bond intermediate, leading to co-elimination of H_2_S and NH_3_ (Watanabe and Snell, 1977). In the CDs catalyzed reaction, the sulfur with a valence of -2 does not undergo oxidoreduction. In the CAT/MST pathway, MST metabolizes MP to pyruvate and produces sulfane sulfur, which is initially in the form of a persulfide at the active site (Toohey, 2011; Yadav et al., 2013); MST can pass the sulfane sulfur to GSH producing GSSH (Figure 3, 4). Although the produced sulfane sulfur can be reduced to H_2_S by thioredoxin and glutaredoxin inside cells (Dóka et al., 2016), the CAT/MST pathway did not clearly contribute to H_2_S production during normal growth (Figure 1D,E) or during the metabolism of exogenous L-cysteine (Figure 1B).

GSSH produced by MST is relatively stable. GSSH was the direct product of MP oxidation by MST in the presence of GSH, and H_2_S was the subsequent reaction product of GSSH with excessive GSH via a relatively slow process (Figure 3, 4). This finding is in agreement with the kinetic analysis of MST for the release of H_2_S from MP in the presence of various small thiols and thioredoxin (Mikami et al., 2011; Yadav et al., 2013). Further, *E. coli* (AtBlh) metabolized MP and produced significant amounts of sulfite and thiosulfate (Figure 3), suggesting that GSSH is an intermediate and is oxidized by the recombinant persulfide dioxygenase AtBlh to sulfite. Sulfite spontaneously reacts with reactive sulfane sulfur to generate thiosulfate (Xin et al., 2017). The results are consistent with the sulfur part of L-cysteine is metabolized by CAT/MST/PDO to thiosulfate in the plant *Arabidopsis thaliana* (Höfler et al., 2016). However, *in vivo* release of H_2_S by *E. coli* cells metabolizing MP was relatively rapid (Figure 4). This is likely because the elevated levels of reactive sulfane sulfur inside *E. coli* cells during MP degradation. The high concentration promotes its reduction by cellular thiols, including GSH, thioredoxin, and glutaredoxin (Fahey et al., 1978; Mclaggan et al., 1990; Westrop et al., 2009; Mikami et al., 2011; Dóka et al., 2016).

CDs are multifunctional enzymes (Han et al., 2001), and L-cysteine metabolism is usually not their main physiological function. For example, TnaA is better known as tryptophanase that degrades L-tryptophan to indole, and it is also important in degrading excessive L-cysteine, as the mutant is inhibited by L-cysteine in the medium (Awano et al., 2005). Our data support the role of TnaA in degrading exogenous L-cysteine (Figure 1C). However, its deletion did not affect H_2_S production during the growth in LB medium (Figure 1D,E). This is expected since TnaA has an apparent Km for L-cysteine of 11 mM(Snell, 1975; Oguri et al., 2012) and L-cysteine is normally around 200 μM in *E coli* (Park and Imlay, 2003). Collectively, the physiological role of CDs is likely to detoxify excess L-cysteine, as previously suggest (Awano et al., 2005). Although CysK and CysM have CD activity (Awano et al., 2005), they are better known as cysteine synthases that consume H_2_S (Kredich and Tomkins, 1966; Zhao et al., 2006). Thus, it is expected to see H_2_S accumulation in *E. coli*Δ*cysK* and *E. coli*Δ*cysK*Δ*cysM* (Figure 1D,E). The same phenomenon has been observed with *Shewanella oneidensis* and its Δ*cysK* mutant produces more H_2_S (Wu et al., 2015). The double mutant *E. coli*Δ*cysK*Δ*cysM* showed a reduced biomass (Figure 1F), likely due to the short supply of L-cysteine. Surprisingly, *E. coli*Δ*cysM* accumulated slightly less H_2_S than the wild type. Perhaps, CysM also uses thiosulfate in LB medium to produce L-cysteine (Zhao et al., 2006), and the extra L-cysteine may contribute to H_2_S production in the wild type.

Nagahara and Sawada report that the CAT/MST pathway dose not contribute much to L-cysteine degradation (Nagahara and Sawada, 2006), which is in agreement with our data that the mutant has a limited impact on L-cysteine degradation and H_2_S production (Figure 1D,E). This is not a surprise, since CAT is also a multifunctional enzyme that also catalyzes the metabolism of aspartate, phenylalanine and tyrosine via transamination (Gelfand and Steinberg, 1977; Han et al., 2001). The ability of the CAT to catalyze the transamination for L-cysteine is much weaker than for aspartate (Sung et al., 1990), implying that L-cysteine is not a preferred substrate for CAT under the physiological conditions. This is also consistent with our observation that *E. coli* Δ*sseA* released similar amount of H_2_S as the wild type when growing in LB medium; the finding is different from a previous observation (Shatalin et al., 2011) that the *sseA* mutant produced less amount of H_2_S. However, our results are consistent with the *sseA* mutant of *S. oneidensis* that produced the same amount of H_2_S as the wild type(Wu et al., 2015).

The deletion of *sseA* has been reported to make the mutant sensitive to H_2_O_2_ (Shatalin et al., 2011) which is in agreement with our results with *E. coli* Δ*sseA*, but they hypothesized that H_2_S was responsible for the resistance. They also provided additional evidence to support the function of H_2_S and proposed H_2_S to sequestrate Fe^2+^ inside the cell to minimize the Fenton’s reaction as a mechanism (Mironov et al., 2017). Since we only observed a reduction in reactive sulfane sulfur, we attributed the antioxidant activity to the latter, which is in line with its chemical and physical properties (Greiner et al., 2013; Ida et al., 2014; Toohey and Cooper, 2014). Reactive sulfane sulfur GSSH and CysSSH are approximately 10∼100 times more reactive toward H_2_O_2_ than GSH and L-cysteine (Saund et al., 2015; Kasamatsu et al., 2016), and GSSH is approximately 50 times more reactive than H_2_S toward H_2_O_2_ (Ida et al., 2014). *E. coli* is known to use different approaches to resist H_2_O_2_, such as the Oxy regulon (Zheng et al., 2001) and the L-cysteine/L-cystine shuttle system (Iwao et al., 2010). Here we presented evidence to support that reactive sulfane sulfur is an additional mechanism.

Bioinformatics data showed that MST is widely distributed in bacteria. About 48.2% sequenced bacterial genomes contain MST, which is significantly more than the 20.6% for SQR (Xia et al., 2017). The wide distribution implies for its important roles such as protecting cells from oxidative stress. MSTs are also present in plants and animals (Höfler et al., 2016; Kuo et al., 2016). The phylogenetic tree of MSTs suggests that they are highly heterogeneous, but clearly separated from rhodaneses that are also highly heterogeneous (Cipollone et al., 2010). Cysteinyl-tRNA synthetase is universal in all organisms, and it can produce reactive sulfane sulfur (Akaike et al., 2017). However, cysteinyl-tRNA synthetase is relatively inefficient in producing Cys-SSH, and only about 4 μM Cys-SSH is generated after it metabolizes 100 μM L-cysteine in 60 min (Akaike et al., 2017). In comparison, MST is a specific enzyme for the production of reactive sulfane sulfur and quickly generating elevated reactive sulfane sulfur (Figure 3A). Thus, cysteinyl-tRNA synthetase may play a significant role in generating low levels of reactive sulfane sulfur, and MST has the ability to produce additional reactive sulfane sulfur, coffering *E. coli* with resistance to oxidative stress.

## Conclusion

**Figure 8:**
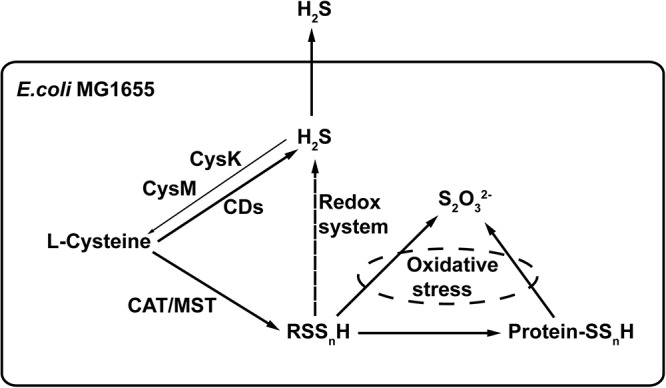
The proposed model of H_2_S and reactive sulfane sulfur production in *E. coli*. CDs convert L-cysteine to H_2_S. CAT/MST metabolize L-cysteine to generate reactive sulfane sulfur that can render protein sulfhydration. Excessive reactive sulfane sulfur is reduced to H_2_S by cellular thiols and thioredoxin/glutaredoxin, but this is not an active route of H_2_S production during normal growth. Although CysK and CysM use H_2_S to produce L-cysteine, most of the produced H_2_S is lost via evaporation. H_2_O_2_ can oxidize reactive sulfane sulfur to thiosulfate (Nagahara et al., 2012).

L-cysteine is the major substrate for the production of H_2_S and reactive sulfane sulfur in *E. coli* (Figure 8). CDs produce H_2_S, and the CAT/MST pathway generates reactive sulfane sulfur. The two forms are not readily interchangeable in *E. coli*, as it cannot easily oxidize H_2_S to reactive sulfane sulfur and the reduction of reactive sulfane sulfur by cellular thiols is not likely a major route for H_2_S production, as *E. coli* Δ*sseA* generated similar amount of H_2_S but contained less sulfane sulfur (Figure 6B,D). The produced H_2_S is mainly lost through evaporation into the gas phase. Therefore, H_2_S is not accumulated inside the cell and its concentration is likely not high enough to be responsible for protein sulfhydration under normal growth conditions. Reactive sulfane sulfur is relatively stable and is constantly present inside cells, and it is likely involved in protein sulfhydration and confers the cell with resistance to oxidative stress. Our findings provide a basis for further investigation of the cellular functions of reactive sulfane sulfur.

## Author Contributions

KL acquired and analyzed the data. YFX and GX contributed to the gene deletion and plasmid construction. RZ helped with the bioinformatics. HL and YZX supervised the research. KL, YZX, and LX designed the study and wrote the manuscript.

## Conflict of Interest Statement

The authors declare that the research was conducted in the absence of any commercial or financial relationships that could be construed as a potential conflict of interest.

## Footnotes

1 https://www.ebi.ac.uk/Tools/msa/clustalo/
