# Supplementary material for: Escherichia coli Uses Separate Enzymes to Produce H2S and Reactive Sulfane Sulfur From L-cysteine
Source: Front Microbiol. 2019 Feb 20;10:298. doi: 10.3389/fmicb.2019.00298 (PMC6401616; doi:10.3389/fmicb.2019.00298)
Supplement: Supplementary file 1 [file Data_Sheet_1.pdf]

Supplementary Materials for

Escherichia coli uses separate enzymes to produce H_2S and reactive sulfane sulfur from L-cysteine

Kai Li[1], Yufeng Xin[3], Guanhua Xuan[1], Rui Zhao[1], Huaiwei Liu[1], Yongzhen Xia[1,*], and Luying Xun[1,2,*]

[1] State Key Laboratory of Microbial Technology, Shandong University, Qingdao, P. R. China.

[2] School of Molecular Biosciences, Washington State University, Pullman, WA, USA.

[3] College of Life Sciences, Qufu Normal University, Qufu, P. R. China.

[*] For correspondence. E-mail: luying_xun@wsu.edu; Tel: +1 (509) 335-2787; Fax: +1 (509) 335-4159; xiayongzhen2002@sdu.edu.cn

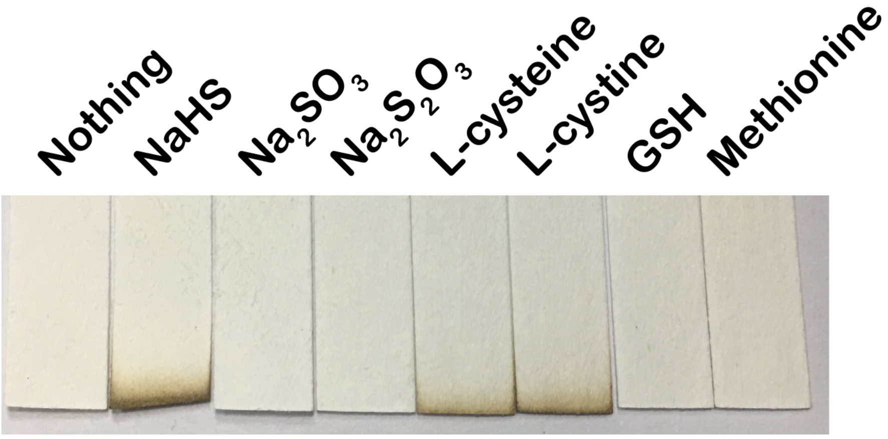

Supplementary Figure S1. H_2S generated from various substrates by resting cells of the wild type in Tris buffer at OD_{600nm} of 2 in 30-min incubation. The production of H_2S was detected by using $Pb(Ac)_2$ paper strips.

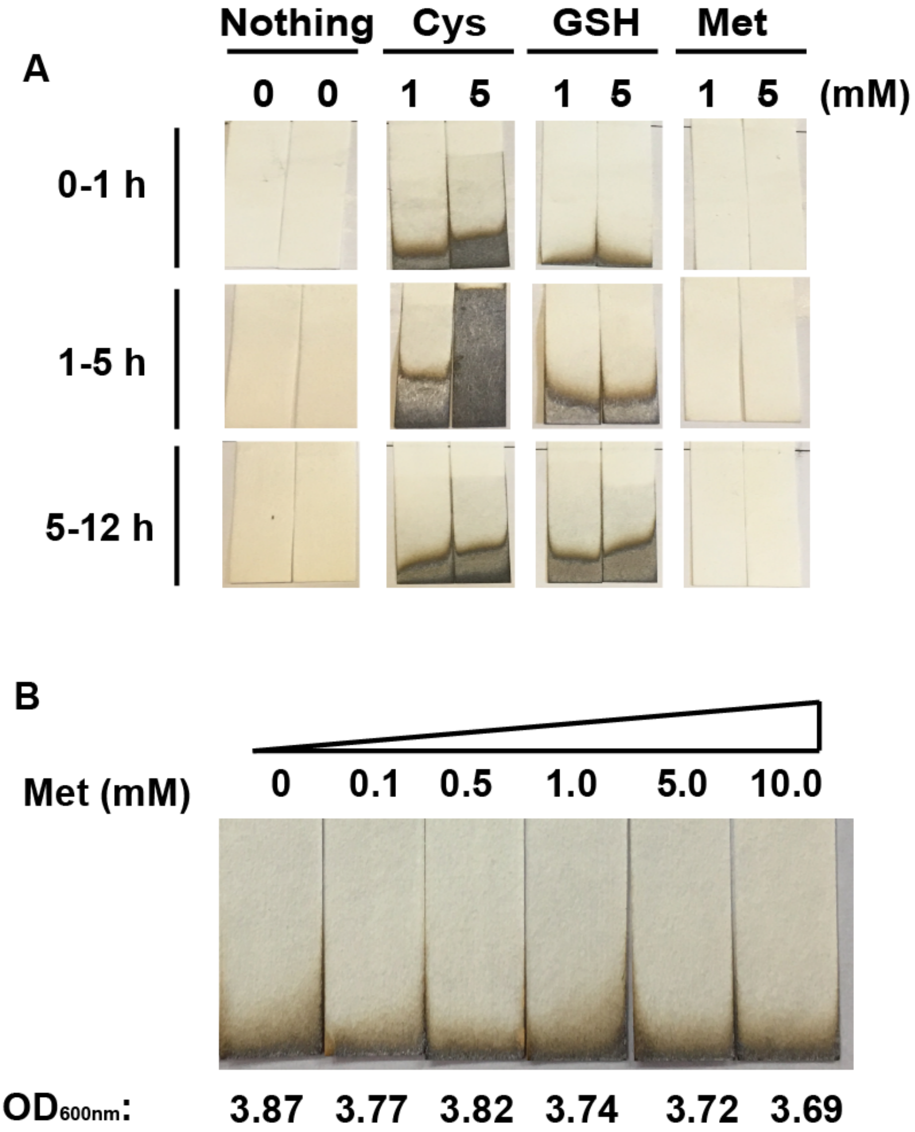

Supplementary Figure S2. The production of H_2S by *E. coli* MG1655 from sulfur-containing compounds. (A) The production of H_2S by *E. coli* MG1655 resting cells from metabolizing L-cysteine (Cys), glutathione (GSH), and methionine (Met) during different incubation periods was detected by using $Pb(Ac)_2$-containing paper strips. **(B)** Overnight cultures of *E. coli* MG1655 were inoculated into LB medium containing different concentrations (0-10 mM) of methionine and incubated at 37°C for 12 h. H_2S production was detected by using $Pb(Ac)_2$-containing paper strips, and the growth yield was estimated with OD_{600nm}.

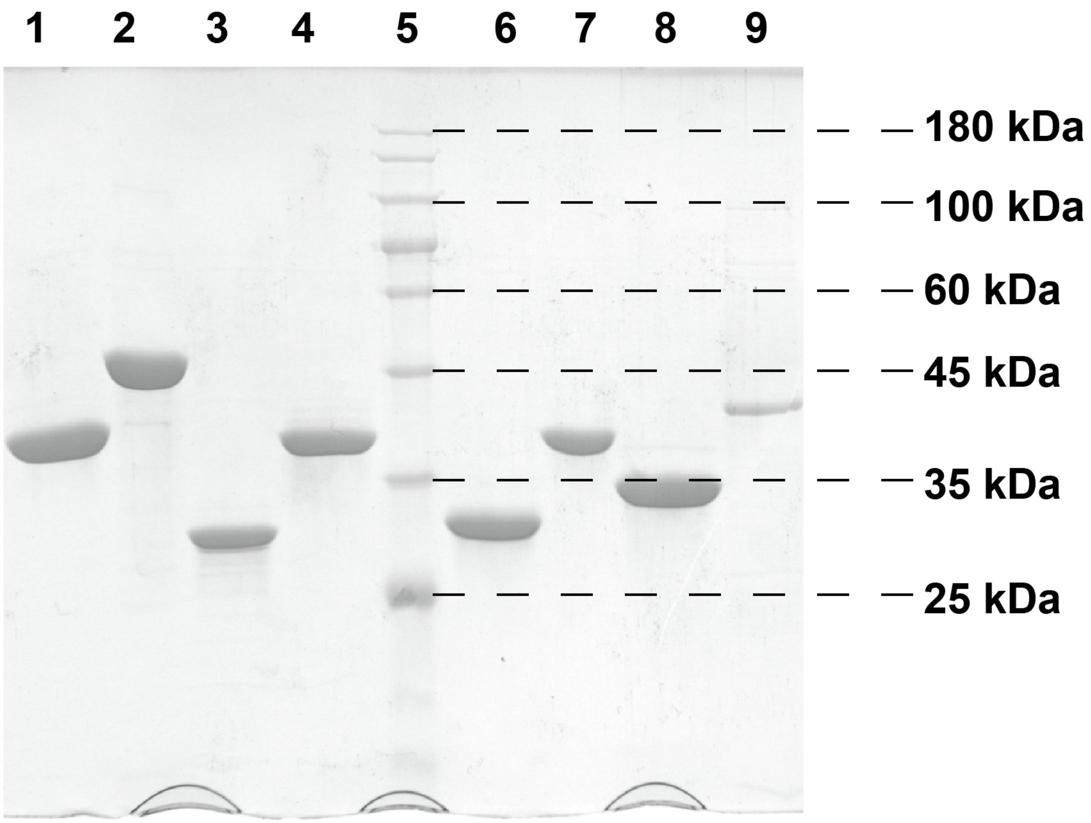

Supplementary Figure S3. The SDS-PAGE of the recombinant proteins. 1, MalY; 2, TnaA; 3, MST; 4, CAT; 5, Marker; 6, CysM; 7, MetC; 8, CysK; 9, YhaM.

Supplementary Table S1. Strains and plasmids used in this study

Plasmid or Strain	Description	source
pET-30 Ec/Lic	Km^r, expression vector	Novagen
pET-TnaA	pET30 Ec/Lic containing *E. coli tnaA*	This study
pET-MetC	pET30 Ec/Lic containing *E. coli metC*	This study
pET-MalY	pET30 Ec/Lic containing *E. coli malY*	This study
pET-CysK	pET30 Ec/Lic containing *E. coli cysK*	This study
pET-cysM	pET30 Ec/Lic containing *E. coli cysM*	This study
pET-yhaM	pET30 Ec/Lic containing *E. coli yhaM*	This study
pET-MST	pET30 Ec/Lic containing *E. coli sseA*	This study
pET-CAT	pET30 Ec/Lic containing *E. coli aspC*	This study
pBBR1MCS2	Km^r, mob^+, pBBR1 replicon, cloning vector	Kovach[a]
pBBR1MCS2-AtBLH	pBBR1MCS2 containing blh from At. C58	This study
E. coli (pBBR2)	*E. coli* MG1655/pBBR1MCS2	This study
E. coli (AtBlh)	*E. coli* MG1655/pBBR1MCS2- AtBlh	This study
E. coli Δ*sseA* (pBBR2)	*E. coli* ΔsseA/pBBR1MCS2	This study
E. coli Δ*sseA* (AtBlh)	*E. coli* ΔsseA/pBBR1MCS2- AtBlh	This study
E. coli Δ*sseA* (MST)	*E. coli* ΔsseA/pBBR1MCS2- MST	This study
E. coli (CAT/MST)	*E. coli* MG1655/pBBR1MCS2- CAT/MST	This study
E. coli Δ*malY*	*malY* deletion of *E. coli* MG1655	This study
E. coli Δ*cysK*	*cysK* deletion of *E. coli* MG1655	This study
E. coli Δ*cysM*	*cysM* deletion of *E. coli* MG1655	This study
E. coli Δ*yhaM*	*yhaM* deletion of *E. coli* MG1655	This study
E. coli Δ*aspC*Δ*sseA*	*aspC* and *sseA* deletion of *E. coli* MG1655	This study

[a] Kovach ME, Elzer PH, Hill DS, Robertson GT, Farris MA, Roop RM 2nd, Peterson KM. 1995. Four new derivatives of the broad-host-range cloning vector pBBR1MCS, carrying different antibiotic-resistance cassettes. Gene. 166:175-176.

Supplementary Table S2. Oligonucleotide primers used for plasmid construction

Name	Sequence
Fr-pET30-*tnaA*	TTAAGAAGGAGATATACATAATGGAAAACTTTAAACATCTCCCTG
Rev-pET30-*tnaA*	TGGTGGTGGTGGTGCTCGAGAACTTCTTTAAGTTTTGCGGTG
Fr-pET30-*malY*	TTAAGAAGGAGATATACATAATGTTCGATTTTTCAAAGG
Rev-pET30-*malY*	TGGTGGTGGTGGTGCTCGAGACGAACAGCGCGGAT
Fr-pET30-*cysM*	TTAAGAAGGAGATATACATAGTGAGTACATTAGAACAAACAATAG
Rev-pET30-*cysM*	TGGTGGTGGTGGTGCTCGAGAATCCCCGCCCCCT
Fr-pET30-*cysK*	TTAAGAAGGAGATATACATAATGAGTAAGATTTTTGAAGATAACT
Rev-pET30-*cysK*	TGGTGGTGGTGGTGCTCGAGCTGTTGCAATTCTTTCTC
Fr-pET30-*metC*	TTAAGAAGGAGATATACATAATGGCGGACAAAAAGCTTG
Rev-pET30-*metC*	TGGTGGTGGTGGTGCTCGAGTACAATTCGCGCAAAACC
F-pET30-*yhaM*	TTAAGAAGGAGATATACATAATGTTTGATTCGACTTTAAATCCGT
Rev-PET30-*yhaM*	TGGTGGTGGTGGTGCTCGAGTCTGGCCTTGCTCGCCATAAT
Fr-pET30-*aspC*	TTAAGAAGGAGATATACATAATGTTTGAGAACATTACCGCC
Rev-pET30-*aspC*	AGTGGTGGTGGTGGTGGTGCTCGAGCAGCACTGCCACAATCG
Fr-pET30-*sseA*	TTAAGAAGGAGATATACATAATGTCCACGACATGGTTTGTAGGAG
Rev-pET30-*sseA*	AGTGGTGGTGGTGGTGGTGCTCGAGTTTCACTGGCTCAACCGGTAAATCT
Fr-pBBR2-AtBIh	CACACAGGAAACAGCTATGAAGGCCGTAAGGATCAACG
Rev-pBBR2-AtBIh	TAACAAAATATTAACGCTCACCATGTGCTGCCCTC
Fr-pBBR2sseA	CACACAGGAAACAGCTATGTCCACGACATGGTTTGTAGGAG
Rev-pBBR2-*sseA*	TAACAAAATATTAACGCTCATTTCACTGGCTCAACCGGTAAATCT
Fr-pBBR2-*aspC*-*sseA*	TAACTTTAAGAAGGAGATATACATAATGTCCACGACATGGTTTGTAGGAG
Rev-pBBR2-*aspC*-*sse*	TATGTATATCTCCTTCTTAAAGTTATTACAGCACT

A	GCCACAATCG

Supplementary Table S3. The distribution of MST containing strains in phyla

Class	Number
Acidimicrobiia	1
Acidobacteriales	2
Acidothermales	1
Actinobacteria incertae sedis	1
Actinomycetales	1
Actinopolysporales	1
**Alphaproteobacteria**	**513**
Anaerolineae	1
Ardenticatenia	1
**Bacilli**	**305**
Bacteroidetes Order II. Incertae sedis	3
Bacteroidia	1
**Betaproteobacteria**	**766**
Caldilineae	1
Candidatus Nanopelagicales	3
Catenulisporales	1
Chitinophagia	3
Chloroflexia	2
Chroococcidiopsidales	1
Clostridia	3
**Corynebacteriales**	**249**
Cytophagia	7
Deinococci	15
Deltaproteobacteria	9
Flavobacteriia	70
Frankiales	5
Fusobacteriales	1
**Gammaproteobacteria**	**1897**
Gemmatimonadales	2
Geodermatophilales	2
Glycomycetales	1
Kineosporiales	1
Limnochordia	1
Micrococcales	76
Micromonosporales	11
Nakamurellales	1
Negativicutes	3
Nostocales	20
Oscillatoriophycideae	25
Planctomycetia	1
Pleurocapsales	3

Propionibacteriales	1
Proteobacteria	4
Pseudonocardiales	30
Rubrobacteria	1
Saprospiria	1
Sphingobacteriia	8
Streptomycetales	81
Streptosporangiales	6
Synechococcales	22
Thermoleophilia	1
unclassified Verrucomicrobia	1
**Total**	**4183**
